# Feasibility of multiplexed gene mutation detection in plasma samples of colorectal cancer patients by mass spectrometric genotyping

**DOI:** 10.1371/journal.pone.0176340

**Published:** 2017-05-01

**Authors:** Su-Jin Shin, Sung-Min Chun, Tae-Im Kim, Yu Jin Kim, Hyun-Jeung Choi, Se Jin Jang, Jihun Kim

**Affiliations:** 1 Department of Pathology, University of Ulsan College of Medicine, Asan Medical Center, Seoul, Republic of Korea; 2 Asan Institute for Life Science, University of Ulsan College of Medicine, Asan Medical Center, Seoul, Republic of Korea; 3 The Center for anti-cancer CDx, N-Bio, Seoul National University, Seoul, Republic of Korea; CNR, ITALY

## Abstract

Mutation analysis of circulating tumor DNA (ctDNA) has recently been introduced as a noninvasive tumor monitoring method. In this study, we tested the mass spectrometric-based MassARRAY platform for multiplexed gene mutation analysis of plasma samples from colorectal cancer (CRC) patients. A total of 160 patients, who underwent curative resection of either primary or metastatic CRC harboring *KRAS* mutations between 2005 and 2012, were included. Circulating DNA was isolated from plasma was analyzed on the MassARRAY platform with or without selective amplification of mutant DNA fragments. Tumor-specific *KRAS* mutations were detected in 39.6% (42/106) of patients with distant metastasis, and in 5.6% (3/54) of patients without distant metastasis. Selective amplification of the mutant allele increased sensitivity to 58.5% (62/106) for patients with distant metastasis, and 16.7% (9/54) for patients without distant metastasis. These mutation detection rates were no less than those of droplet digital polymerase chain reaction. Among patients with distant metastasis, detectable plasma *KRAS* mutations correlated with larger primary tumors and shorter overall survival rate (*P* = 0.014 and *P* = 0.003, respectively). In addition, activating *PIK3CA* mutations were detected together with *KRAS* mutations in two plasma samples. Taken together, massARRAY platform is a cost-effective, multigene mutation profiling technique for ctDNA with reasonable sensitivity and specificity.

## Introduction

Colorectal cancer (CRC) is the third most common cancer and the major cause of cancer-related mortality in South Korea [1]. Activating mutations in the RAS family genes, such as *KRAS*, *NRAS*, and *BRAF*, are known to be associated with resistance to anti-EGFR monoclonal antibodies, such as cetuximab or pannitumumab [2–5]. *KRAS* mutations can also be acquired during anti-EGFR therapy, leading to regrowth of initially *KRAS* wild type CRCs [6–8]. Detection of mutations that lead to acquired resistance to anti-EGFR therapy is clinically important but is limited in most cases by the unavailability of the metastatic tumor tissue. Thus, the analysis of circulating tumor markers, such as of circulating tumor DNA (ctDNA) and circulating tumor cells (CTCs), has emerged as an attractive alternative.

Recently, CTCs are known to predict prognosis of metastatic CRC patients [9, 10] and various genomic analysis techniques have been tried on isolated CTCs. However, the utility of CTCs is limited because of low CTC numbers. Only 52.5% of advanced cancer patients had more than three tumor cells per 7.5 mL blood and the mutational concordance rate between CTCs and the primary tumor tissue was only 50% [11]. As another circulating tumor marker, ctDNA is known to be useful for investigating intratumoral heterogeneity, prognosis and prediction of treatment response [12]. CtDNA refers to extracellular DNA fragments in plasma that are released by malignant neoplasms [13–15] and its levels are known to correlate with the total tumor burden [16]. Therefore, it has been suggested that ctDNA could be used as a potential marker of residual disease or recurrence [17].

Unfortunately, the detection of tumor-specific mutations in ctDNA requires extremely sensitive methods because of the low amount of ctDNA in patients’ plasma [16, 18, 19]. Therefore, standard sequencing approaches, including Sanger sequencing or pyrosequencing, are not sufficiently sensitive [17] and only extremely sensitive methods such as digital polymerase-chain-reaction (PCR) or tagged amplicon deep sequencing could successfully detect mutations in ctDNA samples [20]. In addition, an ideal ctDNA analysis technique should be able to detect genetic evolution of tumors over the treatment course. To do this, multiple genes should be analyzed in parallel. Ultra-deep NGS can achieve both sufficiently high sensitivity and multiplexing, it costs too much.

Matrix-assisted laser desorption/ ionization (MALDI) time-of-flight (TOF) mass spectrometry (MS) combined with iPLEX GOLD chemistry has facilitated higher-throughput analysis of SNPs [21, 22]. Since this technology can easily analyze up to 40 SNPs in a single reaction with reasonable sensitivity and specificity and can give relative abundance of each SNP [22], we evaluated the clinical utility of this platform as a non-invasive method for CRC monitoring by analyzing plasma samples from CRC patients whose tumors have known *KRAS* mutations by using a multigene format MALDI-TOF MS platform.

## Materials and methods

### Patients and samples

A total of 160 participants were selected from patients who underwent curative surgical resection of primary and/or metastatic CRC at the Asan Medical Center (AMC) between 2005 and 2012. All resected tumors were pathologically confirmed adenocarcinomas harboring *KRAS* mutations, detected by Sanger sequencing. No patient received pre-operative chemotherapy or radiotherapy. Among 106 patients with distant metastases at the time of initial plasma sampling, 103 patients had distant metastases at the time of diagnosis and had primary tumors and metastases resected at the same time. The remaining three patients did not have distant metastases at the time of diagnosis but later developed metachronous metastases, and plasma samples were obtained upon the diagnosis of distant metastases. The 54 patients without distant metastases underwent curative surgical resection only for the primary CRC (Table A in S1 File). Medical records were reviewed for clinical, radiological, laboratory, and pathological findings and clinical outcomes.

From the 160 patients enrolled in this study, 177 plasma samples were collected. Among these samples, 157 samples were obtained on the day before initial surgical resection of the primary CRC with or without metastasectomy and three samples were collected on the day before metachronous metastasectomy. In 17 patients, plasma samples were again obtained at a follow-up visit after the initial diagnosis. All plasma samples in this study were collected, stored, and provided by the Asan Bio-Resource Center, Korea Biobank Network (2014–14 (83)). For non-tumor controls, blood samples were collected from 17 healthy volunteers. Healthy volunteers comprised eight men and nine women, with a mean age of 34 years (range, 26~48 years). Study protocols were compliant with the World Medical Association Declaration of Helsinki recommendations and were approved by the Institutional Review Board of AMC (S2014-0860-0002). Since we used plasma samples that had been collected and stored by the Asan Bio-Resource Center, all CRC patients agreed that they donate their samples to the Bio-Resource Center by signing a written consent form. All written consent forms were securely stored in the Bio-Resource Center and a copy of the original form was given to each patient. We received CRC patient plasma samples from the Bio Resource Center by submitting the Institutional Review Board approval protocols. For healthy volunteers, we obtained a separate written informed consent whose form had been approved by the Institutional Review Board. The signed consent forms are securely stored in the researcher’s locker.

### Processing of plasma

For each enrolled patient, 4 mL of blood was collected in EDTA-containing tubes, and sent to and processed at the Asan Bio-Resource Center 1 h after collection. The supernatant was collected and centrifuged at 1,520 × *g* for 5 min at 4°C to separate plasma from peripheral blood cells. Plasma were samples were transferred to several 1.5-mL tubes and stored at -80°C at the Asan Bio-Resource Center, South Korea. We obtained 200-μL aliquots of blood plasma from the Asan Bio-Resource.

Blood samples of healthy volunteers were also collected in EDTA-containing tubes. Within 1 h after collection, 4 mL of blood was centrifuged at 1,520 × *g* at 4°C for 10 min, and the supernatant was centrifuged again at 16,000 × *g* at 4°C for 10 min. The clear supernatant was stored at -80°C until DNA extraction.

### Extraction of circulating DNA

Circulating cell-free DNA (cfDNA) was extracted from 200 μL of plasma, using QIAamp Circulating Nucleic Acid Kit (Qiagen), following the manufacturer’s instructions. Extracted DNA was quantified using Quant-iT^™^ PicoGreen dsDNA Assay kit (Invitrogen, Carlsbad, CA), and kept at −20°C until use.

### Mutation analysis by MassARRAY iPLEX system

For total cfDNA samples, multiplex mutation analysis was performed using the Asan Colon Panel Version 2.0 (OncoMap_C1) on the Sequenom MassARRAY and iPLEX-Pro chemistry technology platform (Sequenom, San Diego, USA). OncoMap_C1 comprises five pools of iPLEX panels for detecting 73 unique mutations in four genes, including *KRAS*, *NRAS*, *BRAF*, and *PIK3CA* (Table B in S1 File). Multiplex PCR amplification was performed using 5 ng of genomic DNA (in 2 μL) per pool and 0.5 U of Taq polymerase (Qiagen), 1.25X PCR buffer, 1.625 mM MgCl_2_, 500 μM deoxynucleotide triphosphates, and 120 nM of primers (Table B in S1 File). The following thermocycler program was used for amplification: 94°C for 15 min, followed by 45 cycles of (94°C for 20 s, 56°C for 30 s, and 72°C for 1 min), and a final extension step of 72°C for 3 min.

After multiplex PCR amplification reactions were completed, residual deoxynucleotides were inactivated by treatment with shrimp alkaline phosphatase (Cat # 10142–2, Sequenom) at 37°C for 40 min and 85°C for 5 min. Single-base extension reactions were then performed in a total reaction volume of 9 μL containing 0.222X iPLEX buffer plus, 0.5X iPLEX termination mix, extension primer mix (0.93 μM: 1.86 μM), and 0.5X iPLEX enzyme (Thermo sequenase), and using the following nested thermocycler programs: 94°C for 30 s, followed by 40 cycles of [94°C for 5 s, 52°C for 5 s, and 80°C for 5 s]. The annealing and extension steps were repeated five times within the 40 cycle program (i.e., 40 × 5 = 200 short cycles), before a final extension step of 3 min at 72°C. After spotting of the desalted product onto a 384-format SpectroCHIP II, spectrum profiles generated by matrix-assisted laser desorption/ionization time-of-flight mass spectrometry were acquired and interpreted using the TYPER 4.0 software (Sequenom). Raw data were further manually reviewed by two independent researchers to reinterpret uncertain calls resulting from clustering artifacts if necessary.

### Mutation analysis by mutant allele-specific enrichment technology

We have developed an ultrahigh sensitive (UHS) assay, which combines modified Amplification Refractory Mutation System (ARMS) and conventional iPLEX chemistry. Selective amplification of shorter mutant-specific amplicons was performed by combining mutant allele-specific primer sets (Table C in S1 File) with common outer primer sets. The two primer sets share the same reverse primer. The sequence at the 3′ end of the mutation-specific primers was designed to be completely complementary to the target mutation sequence (e.g., GT-3′ for G12V and GA-3′ for G12D), and a single mismatch sequence was introduced at a position 2 bp upstream of the 3′ end to prevent non-specific allele amplification. Subsequent procedures, including shrimp alkaline phosphatase treatment and single-base extension, were identical to those performed with conventional iPLEX chemistry. To determine the gain in sensitivity provided by the UHS method, we examined specific *KRAS* mutations that occurred with high frequency (G13D and G12D/V) in CRC patients (*n* = 135).

### Determination of detection limits of conventional iPLEX and UHS methods

Genomic DNA from H1975 carrying a defined *EGFR* L858R mutation was serially diluted into Beas2B (wild type control) genomic DNA to the following mutant-to-wild type allele fractions: 100%, 10%, 5%, 1%, 0.5%, 0.1% and 0%. Five ng of each diluted DNA sample was used to estimate the analytical detection limit of the UHS assay, and compared to that of conventional iPLEX chemistry using the same sample.

### Mutation analysis by digital droplet PCR

To investigate whether the relatively low tumor-specific mutation recovery rate is attributable to technical limitation or to pre-analytical issues such as actual scarcity of tumor DNA in patients’ plasma, we performed droplet digital PCR analysis in 23 plasma samples with available leftover. We used the Droplet Digital PCR System Bio-Rad, Hercules, CA, USA, http://www.bio-rad.com) which generates 20,000 droplets by water-oil emulsion droplet technology and PCR amplification occurs in each individual droplet. ddPCR reagents were prepared by mixing 20 μL include the specific primer, probes labeled with FAM and HEX reporter fluorophores, QX200 ddPCR superrmix, and template DNA. The mixture was subjected to 20,000 nanoliter-sized droplet used the QX200 Droplet generator (Bio-Rad), followed by PCR with the following parameters: 95°C × 10 minutes (1 cycle); 40 cycles of 94°C × 30 s and 60°C × 1 minute; 98°C × 10 minutes. After thermal cycling, plates were transferred to a QX200 droplet reader (Bio-Rad) and the fluorescence signal of each individual droplet was measured in two optical channels. Each droplet in samples were plotted the graph of fluorescence intensity vs. droplet number using the QuantaSoft v1.7.4 software (Bio-Rad). The number of positive droplets above the threshold was calculated in units of copies/ μL input DNA by Poisson Algorithm.

### Cost analysis of various mutation profiling techniques

To assess the potential economical benefit of our MassARRAY-based method, relative costs of various mutation profiling techniques, including out MassARRAY iPLEX system, ddPCR, and ultra-deep NGS, were compared. Since each method has its own throughput, i.e. a 384 well plate for MassARRAY, a flow-cell for ultra-deep NGS, and a 96 well plate for a ddPCR run, costs were calculated according to the most efficient testing environment where a test run accommodates as many samples as possible. All primers, reagents, library preparation kits, and other consumables were included for cost analyses. For calculation of NGS cost, we assumed that the Illumina platform with amplicon-based target enrichment will be used. Since the extended RAS testing which includes all hotspot mutations involving *KRAS*, *NRAS*, *PIK3CA*, and *BRAF* is used to assess potential candidates for anti-EGFR therapy in CRC, we took those genomic regions as analysis target of MassARRAY or ultra-deep NGS. For ddPCR, only hotspot mutations involving *KRAS* were included considering typical throughput of ddPCR technology. All other costs, such as equipment maintenance fee or depreciation, labor costs, or costs for other non-consumables, were excluded for analysis. Costs for DNA extraction were also excluded.

### Statistical analysis

Statistical analyses were performed using SPSS software (version 18.0; SPSS, Chicago, IL, USA). Differences between groups were compared by either the χ^2^ test or Fisher’s exact test, and Student’s *t*-test or Mann-Whitney U test. The Kaplan-Meier method with log-rank test and multivariate Cox proportional hazards regression models were applied for survival analyses. Two-sided *P*-values < 0.05 were considered statistically significant.

## Results

### Patient characteristics

All 160 CRC patients had *KRAS* mutations in their surgically resected CRC tissue samples, and all those mutations were located at well-characterized hotspot positions. The most common *KRAS* mutation was G12D (58/160, 36.3%), followed by G12V (42/160, 26.3%) and G13D (35/160, 21.9%). The relative frequencies of *KRAS* mutation types were not different between cases with distant metastasis at the time of diagnosis and those without distant metastasis (Table D in S1 File). Patients in the non-metastasis group lived longer than those in the metastasis group (median survival time: 70.3 months vs 26.1 months, respectively, *P* <0.001).

### Concentrations of circulating DNA in plasma

**Fig 1:**
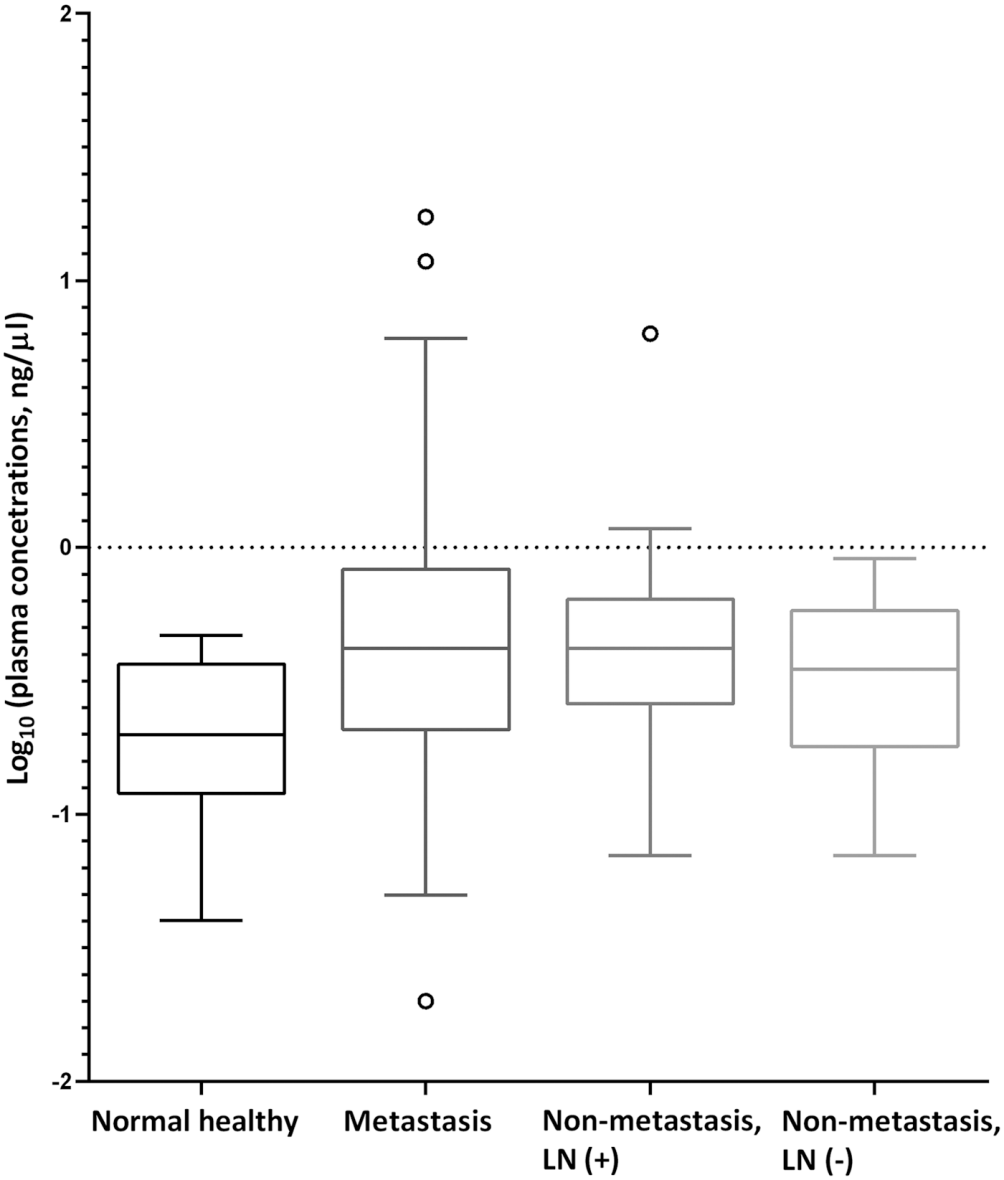
Box and whisker plots of concentrations of circulating DNA. The concentration of DNA was significantly higher in patient groups (metastasis and non-metastasis groups) than in healthy volunteers (*P*<0.001). There was no significant difference between patient groups. (Horizontal line in the middle of each box, median; boxes, 25 percentile ~ 75 percentile; whiskers, 1.5 x interquartile range from each boundary of the boxes; circles, outlier values with corresponding case number; *P*-value by Mann-Whitney U test, two-tailed).

CRC patients, both metastasis and non-metastasis groups, had higher plasma DNA concentrations than healthy volunteers (*P*<0.001; Mann-Whitney U test) and patients in the metastasis group had a tendency towards higher plasma DNA concentrations than those in the non-metastasis group (*P* = 0.277, Mann-Whitney U test). Plasma DNA concentrations were not affected by the presence of regional lymph node metastasis (*P* = 0.463, Mann-Whitney U test) (Fig 1).

### Detection of tumor-specific mutations in circulating DNA by multi-gene MassARRAY analysis with iPLEX chemistry

Among the 160 patients with known *KRAS* mutations in the resected CRC tissue, we detected tumor-specific *KRAS* mutations in the plasma of 39.6% (42/106) of metastasis group and 5.6% (3/54) of the non-metastasis group. All *KRAS* mutations detected in plasma samples were concordant with those confirmed in patient-matched tumor tissue samples. In addition, both the *PIK3CA* H1047R and *KRAS* G12D mutations were detected together in two samples. In contrast, no mutations were detected in plasma samples from the 17 healthy control subjects (Table E in S1 File).

### Increased sensitivity with ultra-high sensitivity assay

**Fig 2:**
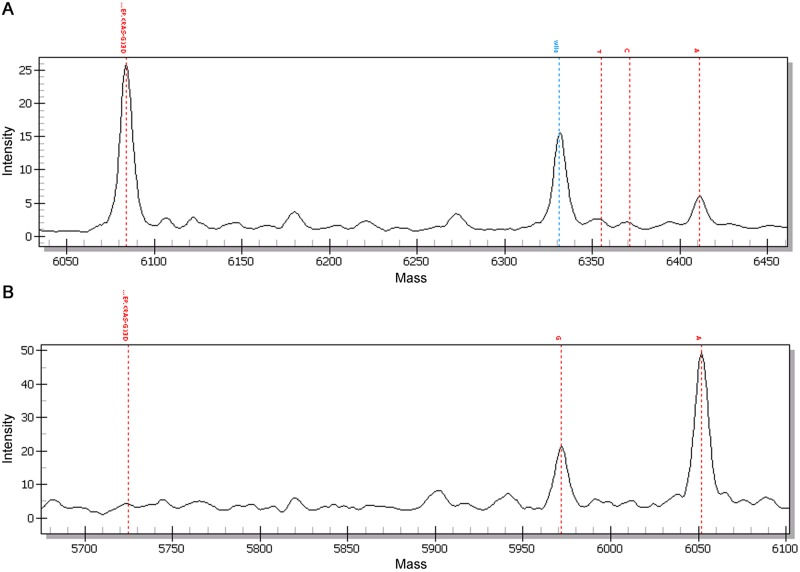
Representative Sequenom outputs. Sequenom output for the *KRAS* G13D mutation is shown. (A) Conventional iPLEX analysis shows a smaller mutant peak revealing relative allelic fractions. (B) The amplitude of the mutant peak is taller in UHS analysis than in conventional iPLEX analysis. (A, adenine; T, thymine; G, guanine; C, cytosine; A on the right side represents the mutant peak and G on the left side represents the wild type peak; the wild type peak in conventional iPLEX analysis is marked with blue color (panel A)).

A total of 135 plasma samples from patients with CRC, whose tissue harbored the *KRAS* G12D/V or G13D mutation, were tested with the ultra-high sensitivity (UHS) method and we could detect tumor-specific *KRAS* mutations in additional 26 plasma samples in which tumor-specific *KRAS* mutations had not been detected in multiplex iPLEX analysis. In addition, signal amplitudes of mutations were higher in the UHS method than in multiplex iPLEX analysis (Fig 2). Consequently, *KRAS* mutations were detected in 58.0% (51/88) of patients in the metastasis group and 14.9% (7/47) of patients in the non-metastasis group (Table 1). *KRAS* mutations detected in plasma were concordant with those confirmed in patient-matched tumor tissue in most cases but, in one patient, *KRAS* G13D mutation was detected in plasma but *KRAS* G12D mutation was in matched tumor tissue. The *KRAS* G13D mutation in the plasma sample was not detected by conventional iPLEX analysis.

**Table 1:** Tumor-specific mutation detection rates in groups classified according to the presence of distant metastasis at the time of blood sampling.

UHS method (n = 135, G12D/V or G13D mutated case)			*P*-value^a^
Plasma *KRAS* mutation	Metastasis group (n = 88)	Non-metastasis group (n = 47)	<0.0001
Detected	51 (58.0%)	7 (14.9%)	
Not detected	37 (42.0%)	40 (85.1%)	
**Combination of iPLEX and UHS method (n = 160)**			
Plasma *KRAS* mutation	Metastasis group (n = 106)	Non-metastasis group (n = 54)	<0.0001
Detected	62 (58.5%)	9 (16.7%)	
Not detected	44 (41.5%)	45 (83.3%)	

^a^ P-values were calculated using the χ^2^ test.

**Fig 3:**
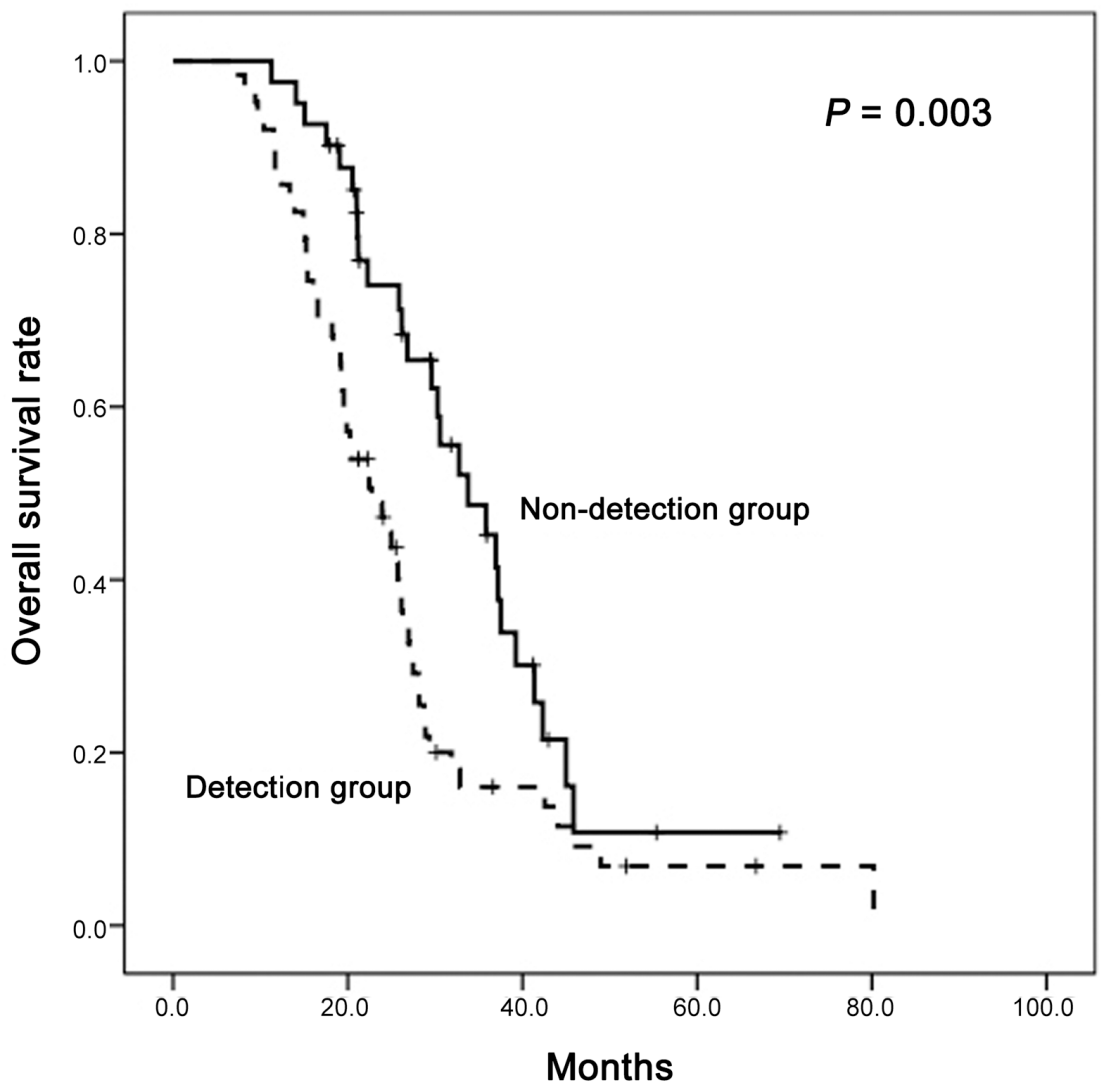
Comparison of survival rates of patients in the metastasis group with or without detectable plasma tumor-specific *KRAS* mutations. Overall survival rates are poorer for patients with detectable plasma *KRAS* mutations than patients without detectable plasma *KRAS* mutations.

The combination of multiplex iPLEX analysis and the UHS method retrieved tumor-specific *KRAS* mutations, which had been found in the patient tumor tissue, in plasma samples from 58.5% (62/106) of metastasis group and 16.7% (9/54) of non-metastasis group (Table 1). The presence of detectable tumor-specific *KRAS* mutations in the plasma correlated with heavier tumor burden. Even within the metastasis group, detectable plasma *KRAS* mutations correlated with larger primary tumor sizes (*P* = 0.014, Table 2) and shorter overall survival rates (*P* = 0.003, Fig 3). Among the 54 patients in the non-metastasis group, patients with detectable plasma *KRAS* mutations developed metachronous metastasis more frequently (8/9, 88.9% vs. 35/45, 77.8%) and at earlier time points (median time to progression: 17.1 months vs. 20.6 months) than those without detectable plasma *KRAS* mutations, although the differences were not statistically significant. (Table F in S1 File). No mutations were detected in plasma samples from the 17 healthy volunteers by the UHS method (Table E in S1 File).

**Table 2:** Comparison of clinicopathological features between patient subgroups with or without detectable plasma tumor-specific *KRAS* mutations within the metastasis group (n = 106).

Clinicopathological features	*KRAS* mutation in plasma		*P*-value
	Detected (n = 62)	Not detected (n = 44)	
**Mean age, years (range)**	57 (27–84)	59 (26–78)	0.302^a^
**Sex**			0.285
**Male**	33 (53.2%)	28 (63.6%)	
**Female**	29 (46.8%)	16 (36.4%)	
**Median primary tumor volume, cm**^**3**^ **(range)**	23.6 (3.02–323.98)	12.6 (1.65–167.55)	0.014^a^
**pT stage**			0.202
**2**	0 (0%)	1 (2.3%)	
**3**	45 (72.6%)	36 (81.8%)	
**4**	17 (27.4%)	7 (15.9%)	
**pN stage**			0.777
**0**	13 (21.0%)	11 (25.0%)	
**1**	31 (50.0%)	19 (43.2%)	
**2**	18 (29.0%)	14 (31.8%)	
**Lymphovascular invasion**			0.786
**Absent**	35 (56.5%)	26 (59.1%)	
**Present**	27 (43.5%)	18 (40.9%)	
**Perineural invasion**			0.786
**Absent**	35 (56.5%)	26 (59.1%)	
**Present**	27 (43.5%)	18 (40.9%)	
**Median plasma concentration, ng/μL (range)**	0.39 (0.05–11.81)	0.43 (0.02–17.28)	0.666^a^
**Multiple metastasis**			0.313
**No (single metastasis)**	48 (78.7%)	38 (86.4%)	
**Yes**	13 (21.3%)	6 (13.6%)	

^a^ P-values were calculated using the Mann-Whitney U test.

### Comparison of sensitivity to digital droplet polymerase chain reaction (ddPCR) assays

Since tumor-specific *KRAS* mutations were detected in only 58.5% of plasma samples from metastatic CRC patients whose tumors had known *KRAS* mutations, we investigated whether this low detection rate was due to technical limitations of our methods or due to low abundance of tumor DNA in plasma. We first determined the analytical sensitivity of our methods by analyzing different fractional mixtures of genomic DNA extracted from cell lines with or without confirmed *EGFR* L858R mutations. The limit of detection was 5% for conventional iPLEX chemistry and 0.1% for UHS assay (S1 Fig). We then analyzed 23 available plasma samples with ddPCR which is known to be the most sensitive method [23, 24]. No additional *KRAS* mutations were detected by ddPCR and the detection rate of tumor-specific mutations in samples, where both MassARRAY and ddPCR analyses were done, was higher with the MassARRAY technique (10/19, 52.6% for iPLEX, 14/19, 73.7% for UHS) than with ddPCR (8/19, 42.1%) (Table G in S1 File).

### Diagnostic utility of the MassARRAY technique in longitudinal follow up plasma samples of CRC patients

Post-resection follow-up blood samples were available for 17 patients. Once again, all patients had known *KRAS* mutations in their resected tumor tissue. Nine patients were in the non-metastasis group and 8 of them (88.9%) developed distant metastases at the time of the second blood sampling. Among 9 patients in non-metastasis group, *KRAS* mutations were newly detected in follow-up plasma samples of 3 patients whose plasma were initially negative for *KRAS* mutations (Table H in S1 File). Among 8 patients in the metastasis group, follow-up samples were obtained at the time of additional curative metastasectomy after cytoreductive chemotherapy or radiotherapy. One patient showed positive conversion of *KRAS* mutations and her disease had considerably worsened at the time of the second blood sampling (Table H in S1 File). Negative conversions of *KRAS* mutations were observed in 4 patients whose tumor burdens were significantly reduced (data not shown).

## Discussion

In the present study, MassARRAY analysis of plasma samples from CRC patients, by either the traditional multiplex iPLEX method or the UHS method, detected tumor-specific mutation with reasonable sensitivity and minimal background noise. The detection of tumor-specific *KRAS* mutations in plasma samples correlated with systemic tumor burden and earlier tumor recurrence. We also detected a coexisting oncogenic mutation, *PIK3CA* H1047R, with a known *KRAS* mutation by the multiplex iPLEX method, demonstrating the potential utility of the MassARRAY technique as a multigene mutation profiling method for plasma samples of CRC patients. Although further validation is required, the MassARRAY technique represents a promising method to monitor tumor burden and genomic changes in CRC.

We detected tumor-specific *KRAS* mutations in plasma samples from 58.5% of stage IV CRCs with known tissue *KRAS* mutations. Although this mutation detection rate was lower than that of a previous report (97% by BEAMing) [25], it was no less than that of ddPCR performed in parallel in our study. Considering high sensitivity of the UHS method shown by our cell line mixing experiment, the low detection rate might be due to pre-analytical issues, including plasma isolation technique and low amount of plasma samples. Since plasma samples distributed by our Bio-resource center had been processed by a one-step centrifugation protocol, which can disrupt white blood cells, considerable non-tumor DNA contamination might have occurred. Furthermore, we used a 200 μL plasma aliquot, which is only 10% of the typical volume used in most published studies [25]. Processing larger amount of plasma with optimized DNA extraction method might improve the detection rate of our assays.

The detection of tumor-specific mutations from low input ctDNA requires a very sensitive method, such as allele-specific quantitative PCR [26], ultra-deep NGS [20, 27], BEAMing [16], or ddPCR [23, 24, 28]. Among them, only ultra-deep NGS offers both sufficient sensitivity and multiplexing but it is prohibitively expensive. In this regards, MassARRAY technology might be a compromise between fair performance and affordable cost (Table I in S1 File). Indeed, we detected a coexisting *PIK3CA* H1047R mutation together with a *KRAS* G12D mutation in two plasma samples, demonstrating its utility as a multi-gene assay.

The detection of tumor-specific *KRAS* mutations in plasma correlated with tumor burden in this study. We also inferred mutant *KRAS* copy number from the relative signal heights in the iPLEX method, and input DNA amount. However, mutant DNA copy numbers did not correlate with the estimated tumor volumes on computed tomography (data not shown). It might be that degrees of tumor DNA shedding into the bloodstream are different among tumors. Thus, quantitation of ctDNA might be useful for longitudinal studies of the same patient. One of the limitations of the MassARRAY technique is that quantitation is only available in the iPLEX mode. Because the UHS method involves selective, biased amplification of the mutant allele, mutant peak heights do not mean mutant allelic fraction.

In one particular case, we detected two different *KRAS* mutations, G13D and G12D, from the same patient: G13D from plasma sample by the UHS method and G12D from patient-matched tumor tissue by Sanger sequencing. Neither of these mutations was detected by the multigene iPLEX method. Triplicate repeats of both analyses with plasma and the patient-matched tumor tissue sample yielded the same result. This result may be attributable to the presence of a subclonal tumor population carrying the G13D mutation, or artifacts associated with cytosine deamination [29].

In conclusion, the MassARRAY platform is a cost-effective, multigene mutation profiling method with reasonable sensitivity and specificity for detecting ctDNA. Since the multigene panel used in this study included all currently known predictors of anti-EGFR therapy resistance, including oncogenic mutations in *KRAS*, *NRAS*, *BRAF* and *PIK3CA* [30], the multigene MassARRAY analysis platform might be useful for monitoring genetic changes in the tumor population of metastatic CRC patients receiving anti-EGFR therapy. Furthermore, the UHS method might be useful for the early detection of relapse in CRC patients whose tumors harbor mutations examined in this study. We propose that MassARRAY analysis of ctDNA represents a promising potential for the non-invasive disease monitoring and molecular tracking of CRC.

## Supporting information

S1 Fig Mutant allele peaks detected in variable fractional mixtures of mutant-to-control samples either by conventional iPLEX method or by the UHS method. UHS assay offers ultra-high sensitivity detection of oncogenic mutations. Serially diluted H1975 gDNA with Beas2B gDNA was used to evaluate the detection sensitivity of conventional iPLEX chemistry (limit of detection: ~5%) and the UHS assay (limit of detection: 0.1~0.5%). Blue and red asterisks indicate signals for the mutant and wild type allele, respectively.
(TIF)

S1 File Supporting tables (Tables A-I). (DOCX)
