# Supplementary figures and images for: Feasibility of multiplexed gene mutation detection in plasma samples of colorectal cancer patients by mass spectrometric genotyping
Source: PLoS One. 2017 May 1;12(5):e0176340. doi: 10.1371/journal.pone.0176340 (PMC5411034; doi:10.1371/journal.pone.0176340)

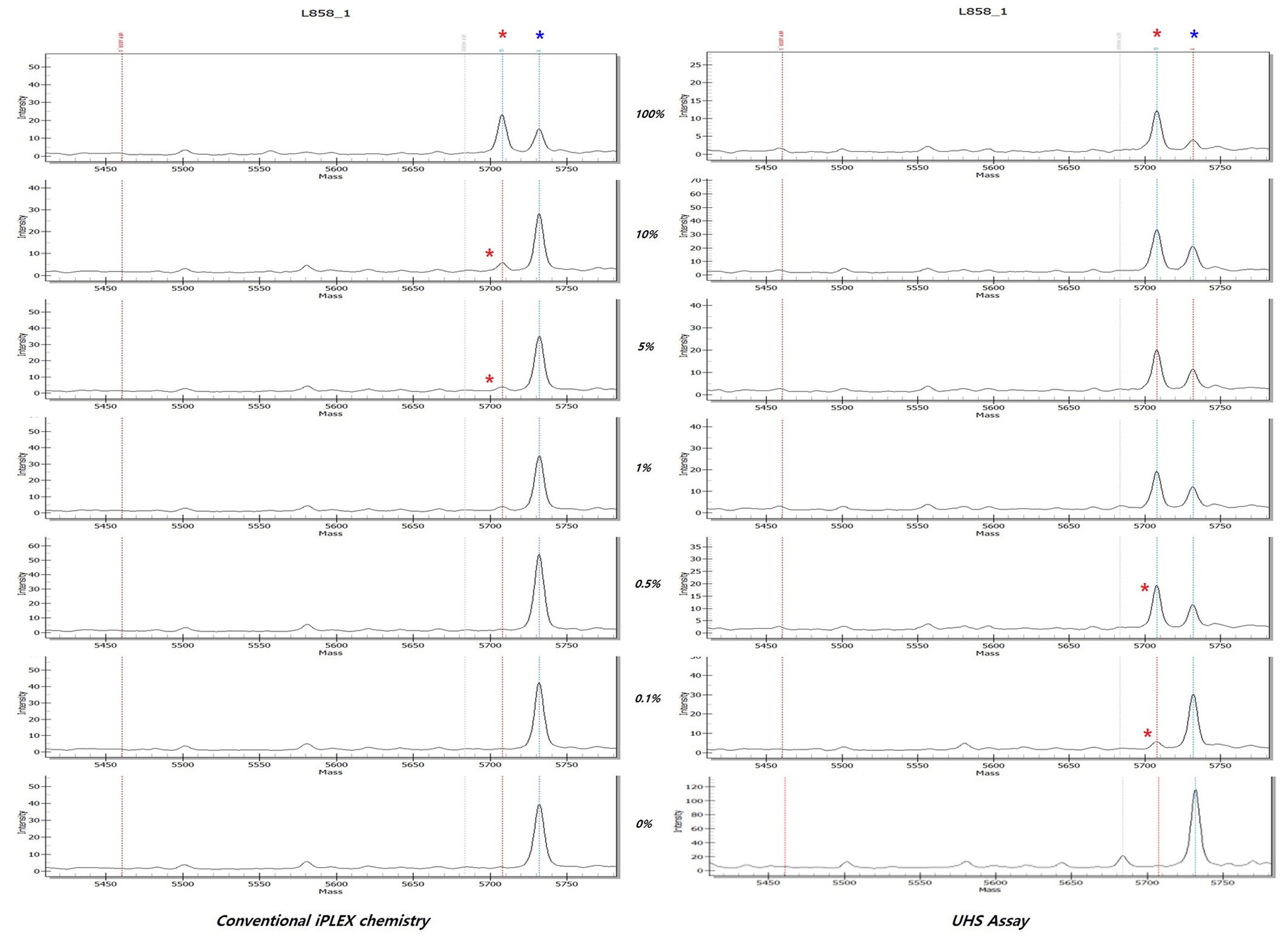

Supplement: S1 Fig — UHS assay offers ultra-high sensitivity detection of oncogenic mutations. Serially diluted H1975 gDNA with Beas2B gDNA was used to evaluate the detection sensitivity of conventional iPLEX chemistry (limit of detection: ~5%) and the UHS assay (limit of detection: 0.1~0.5%). Blue and red asterisks indicate signals for the mutant and wild type allele, respectively. (TIF) [file pone.0176340.s001.tif]
