# Supplementary material for: Feasibility of multiplexed gene mutation detection in plasma samples of colorectal cancer patients by mass spectrometric genotyping
Source: PLoS One. 2017 May 1;12(5):e0176340. doi: 10.1371/journal.pone.0176340 (PMC5411034; doi:10.1371/journal.pone.0176340)
Supplement: S1 File — (DOCX) [file pone.0176340.s002.docx]

**Table A in S1 File. Summary of Patients Group Assignment**

| Metastasis group (n=106) : colorectal adenocarcinoma with distance metastasis | number |
| --- | --- |
| Blood sampling at primary colon cancer surgery | 103 |
| Blood sampling at metastasectomy | 3 |
| Non-metastasis group (n=54) : colorectal adenocarcinoma without distance metastasis |  |
| Presence of LN metastasis, blood sampling at primary colon cancer surgery | 35 |
| Absence of LN metastasis, blood sampling at primary colon cancer surgery | 19 |

Abbreviations: LN, lymph node

**Table B in S1 File. Five Gene-Panels of ASAN Colon Panel Version 2.0 and Their Primers (sequence 5'-3')**

| **Panel** | **SNP_ID** | **PCR Primer_Forward** | **PCR Primer_Reverse** | **Maximum Amplicon Length (bases)** | **Primer for post-PCR extend reaction** |
| --- | --- | --- | --- | --- | --- |
| 1 | KRAS_Q61K/E | ACGTTGGATGCATGTACTGGTCCCTCATTG | ACGTTGGATGTGGAGAAACCTGTCTCTTGG | 96 | TGCACTGTACTCCTCTT (Reverse) |
| 1 | NRAS_A146T/P | ACGTTGGATGGCTGAAAGCTGTACCATACC | ACGTTGGATGACGAACTGGCCAAGAGTTAC | 98 | TACCTGTCTGGTCTTGG (Reverse) |
| 1 | KRAS_A146G/V | ACGTTGGATGTTCAGTGTTACTTACCTGTC | ACGTTGGATGGCTCAGGACTTAGCAAGAAG | 98 | ACTTACCTGTCTTGTCTTT (Reverse) |
| 1 | KRAS_K117N | ACGTTGGATGAGTCCTGAGCCTGTTTTGTG | ACGTTGGATGTGAAGATGTACCTATGGTCC | 100 | TTCTAGAAGGCAAATCACA (Reverse) |
| 1 | PIK3CA_P539R | ACGTTGGATGTTCTCCTGCTCAGTGATTTC | ACGTTGGATGCAGACTAGCTAGAGACAATG | 119 | CTCAGTGATTTCAGAGAGA (Reverse) |
| 1 | NRAS_Q61H | ACGTTGGATGTCGCCTGTCCTCATGTATTG | ACGTTGGATGCCTGTTTGTTGGACATACTG | 99 | CTCATGGCACTGTACTCTTC (Reverse) |
| 1 | KRAS_K117E | ACGTTGGATGTGAAGATGTACCTATGGTCC | ACGTTGGATGAGTCCTGAGCCTGTTTTGTG | 100 | CTATGGTCCTAGTAGGAAAT (Reverse) |
| 1 | PIK3CA_K111E | ACGTTGGATGGTATCATACCAATTTCTCG | ACGTTGGATGACCTTCGGCTTTTTCAACCC | 110 | CAATTTCTCGATTGAGGATCT (Forward) |
| 1 | PIK3CA_D1029H/Y | ACGTTGGATGTACTCCAAAGCCTCTTGCTC | ACGTTGGATGCTCTGGAATGCCAGAACTAC | 113 | CACGCCTCTTGCTCAGTTTTAT (Reverse) |
| 1 | BRAF_V600E | ACGTTGGATGTCTTCATGAAGACCTCACAG | ACGTTGGATGTTCAAACTGATGGGACCCAC | 105 | GGTGATTTTGGTCTAGCTACAG (Forward) |
| 1 | KRAS_A146T/P | ACGTTGGATGTTCAGTGTTACTTACCTGTC | ACGTTGGATGGCTCAGGACTTAGCAAGAAG | 98 | GAATTCCTTTTATTGAAACATCA (Forward) |
| 2 | KRAS_Q61L/P/R | ACGTTGGATGCATGTACTGGTCCCTCATTG | ACGTTGGATGTGGAGAAACCTGTCTCTTGG | 96 | TTGCACTGTACTCCTCT (Reverse) |
| 2 | KRAS_G13C/R/S | ACGTTGGATGAGGCCTGCTGAAAATGACTG | ACGTTGGATGTAGCTGTATCGTCAAGGCAC | 102 | AGGCACTCTTGCCTACGC (Reverse) |
| 2 | NRAS_Q61K/E | ACGTTGGATGTCGCCTGTCCTCATGTATTG | ACGTTGGATGCCTGTTTGTTGGACATACTG | 99 | CATGGCACTGTACTCTTCTT (Reverse) |
| 2 | PIK3CA_E545Q/K | ACGTTGGATGTAGCACTTACCTGTGACTCC | ACGTTGGATGTACACGAGATCCTCTCTCTG | 90 | CATAGAAAATCTTTCTCCTGCT (Reverse) |
| 2 | PIK3CA_M1043I | ACGTTGGATGAACTGAGCAAGAGGCTTTGG | ACGTTGGATGTCCATTTTTGTTGTCCAGCC | 98 | TGGAGTATTTCATGAAACAAAT (Reverse) |
| 2 | NRAS_G13A/D/V | ACGTTGGATGAGTGGTTCTGGATTAGCTGG | ACGTTGGATGGACTGAGTACAAACTGGTGG | 100 | GTGTAAGTGCGCTTTTCCCAACA (Reverse) |
| 3 | NRAS_G13C/R/S | ACGTTGGATGAGTGGTTCTGGATTAGCTGG | ACGTTGGATGGACTGAGTACAAACTGGTGG | 100 | TGCGCTTTTCCCAACAC (Reverse) |
| 3 | PIK3CA_D1029E | ACGTTGGATGTACTCCAAAGCCTCTTGCTC | ACGTTGGATGCTCTGGAATGCCAGAACTAC | 113 | GGCCTCTTGCTCAGTTTT (Reverse) |
| 3 | KRAS_G13A/D/V | ACGTTGGATGAGGCCTGCTGAAAATGACTG | ACGTTGGATGTAGCTGTATCGTCAAGGCAC | 102 | TGGTAGTTGGAGCTGGTG (Forward) |
| 3 | NRAS_Q61L/P/R | ACGTTGGATGCCTGTTTGTTGGACATACTG | ACGTTGGATGTCGCCTGTCCTCATGTATTG | 99 | ATACTGGATACAGCTGGAC (Forward) |
| 3 | PIK3CA_Q546K/E | ACGTTGGATGTAGCACTTACCTGTGACTCC | ACGTTGGATGTACACGAGATCCTCTCTCTG | 90 | TCCATAGAAAATCTTTCTCCT (Reverse) |
| 3 | PIK3CA_P18L | ACGTTGGATGCGGAGGCATTCTAAAGTCAC | ACGTTGGATGATCATCAGGTGAACTGTGGG | 119 | GTAAACATTCTACTAGGATTCTT (Reverse) |
| 4 | KRAS_G12C/R/S | ACGTTGGATGTAGCTGTATCGTCAAGGCAC | ACGTTGGATGAGGCCTGCTGAAAATGACTG | 102 | ACTCTTGCCTACGCCAC (Reverse) |
| 4 | PIK3CA_K111R | ACGTTGGATGACCTTCGGCTTTTTCAACCC | ACGTTGGATGGTATCATACCAATTTCTCG | 110 | AGGCAACCGTGAAGAAA (Forward) |
| 4 | PIK3CA_E542Q/K | ACGTTGGATGTAGCACTTACCTGTGACTCC | ACGTTGGATGGCAATTTCTACACGAGATCC | 98 | TCTCCTGCTCAGTGATTT (Reverse) |
| 4 | NRAS_G12A/D/V | ACGTTGGATGAGTGGTTCTGGATTAGCTGG | ACGTTGGATGGACTGAGTACAAACTGGTGG | 100 | CTCGCTTTTCCCAACACCA (Reverse) |
| 4 | KRAS_Q61H | ACGTTGGATGTGGAGAAACCTGTCTCTTGG | ACGTTGGATGCATGTACTGGTCCCTCATTG | 96 | TATTCTCGACACAGCAGGTCA (Forward) |
| 4 | PIK3CA_T1025A/S | ACGTTGGATGCTCTGGAATGCCAGAACTAC | ACGTTGGATGTACTCCAAAGCCTCTTGCTC | 113 | GAACATTGCATACATTCGAAAG (Forward) |
| 5 | KRAS_G12A/D/V | ACGTTGGATGTAGCTGTATCGTCAAGGCAC | ACGTTGGATGAGGCCTGCTGAAAATGACTG | 102 | CACTCTTGCCTACGCCA (Reverse) |
| 5 | PIK3CA_H1047L/R | ACGTTGGATGTCCATTTTTGTTGTCCAGCC | ACGTTGGATGAACTGAGCAAGAGGCTTTGG | 98 | ATTGTCCAGCCACCATGA (Reverse) |
| 5 | NRAS_G12C/R/S | ACGTTGGATGAGTGGTTCTGGATTAGCTGG | ACGTTGGATGGACTGAGTACAAACTGGTGG | 100 | TAGCTTTTCCCAACACCAC (Reverse) |
| 5 | PIK3CA_E545A/G/V | ACGTTGGATGTACACGAGATCCTCTCTCTG | ACGTTGGATGTAGCACTTACCTGTGACTCC | 90 | ATCCTCTCTCTGAAATCACTG (Forward) |
| 5 | PIK3CA_K111N | ACGTTGGATGGTATCATACCAATTTCTCG | ACGTTGGATGACCTTCGGCTTTTTCAACCC | 110 | GGCAATTTCTCGATTGAGGAT (Reverse) |
| 5 | PIK3CA_M1043V | ACGTTGGATGAACTGAGCAAGAGGCTTTGG | ACGTTGGATGTCCATTTTTGTTGTCCAGCC | 98 | CCCTGGAGTATTTCATGAAACAA (Forward) |

**Table C in S1 File. Primers for UHS Method (Sequence 5'-3')**

| **SNP_ID** | **PCR Primer_Forward** | **PCR Primer_Reverse** | **Mismatch Nested_Forward** | **Nested_Reverse** | **Maximum Amplicon Length (bases)** | **Primer for post-PCR extend reaction** | **Analyte**  **Mass 1 (Da)** | **Analyte**  **Mass 2 (Da)** | **Analyte**  **Mass 3 (Da)** |
| --- | --- | --- | --- | --- | --- | --- | --- | --- | --- |
| G12D/V | ACGTTGGATGTAGCTGTATCGTCAAGGCAC | ACGTTGGATGAGGCCTGCTGAAAATGACTG | ACTTGTGGTAGTTGGAGCAGT (G12V) | TAGCTGTATCGTCAAGGCAC | 102 | CACTCTTGCCTACGCCA (Reverse) | 5313.5 (wild) | 5337.5 (G12V) | 5393.4 (G12D) |
|  |  |  | ACTTGTGGTAGTTGGAGCAGA (G12D) |  |  |  |  |  |  |
| G13D | ACGTTGGATGTAGCTGTATCGTCAAGGCAC | ACGTTGGATGAGGCCTGCTGAAAATGACTG | TGTGGTAGTTGGAGCTGGAGA | TAGCTGTATCGTCAAGGCAC | 102 | CCAGGCACTCTTGCCTACG (Reverse) | 5971.9 (wild) | 6051.8 (G13D) |  |

**Table D in S1 File. *KRAS* Mutation Types of Tumor Tissue in Patient Groups**

| Mutation types | Metastasis group (n=106) | Non-metastasis group (n=54) | |
| --- | --- | --- | --- |
|  |  | LN metastasis (+)  (n=35) | LN metastasis (-)  (n=19) |
| G12A | 7 (6.6%) | 1 (2.9%) |  |
| G12C | 4 (3.8%) | 1 (2.9%) | 4 (21.1%) |
| G12D | 36 (34.0%) | 14 (40.0%) | 8 (42.1%) |
| G12S | 6 (5.7%) | 1 (2.9%) |  |
| G12V | 32 (30.2%) | 7 (20.0%) | 3 (15.8%) |
| G13D | 20 (18.9%) | 11 (31.4%) | 4 (21.1%) |
| G13R | 1 (0.9%) |  |  |

Abbreviations: LN, lymph node

**Table E in S1 File. The Concentration of Circulating DNA and the Results of Mutation Analysis of Healthy Individual Plasma Samples**

| Sample | Age | Sex | Concentration (ng/μl) | Mutation status |
| --- | --- | --- | --- | --- |
| 1 | 36 | M | 0.117 | Non mutated |
| 2 | 35 | F | 0.117 | Non mutated |
| 3 | 29 | F | 0.040 | Non mutated |
| 4 | 27 | M | 0.055 | Non mutated |
| 5 | 28 | F | 0.223 | Non mutated |
| 6 | 26 | F | 0.050 | Non mutated |
| 7 | 29 | M | 0.055 | Non mutated |
| 8 | 29 | M | 0.120 | Non mutated |
| 9 | 40 | M | 0.140 | Non mutated |
| 10 | 29 | F | 0.273 | Non mutated |
| 11 | 43 | F | 0.181 | Non mutated |
| 12 | 29 | M | 0.399 | Non mutated |
| 13 | 47 | M | 0.421 | Non mutated |
| 14 | 35 | F | 0.386 | Non mutated |
| 15 | 33 | M | 0.382 | Non mutated |
| 16 | 48 | F | 0.364 | Non mutated |
| 17 | 32 | F | 0.341 | Non mutated |

**Table F in S1 File. Clinical Characteristics of Non-metastasis Group with Detectable *KRAS* Mutations in the Initial Plasma Sample (n=9)**

| Case NO. | Age | Sex | Mutation type, tissue | Mutation type, plasma (detection method) | cfDNA concentration (ng/μl) | pT stage | Initial tumor size (cm ) | pN stage | LVI | PNI | DFS time (m) | Metastisis site | Survival status |
| --- | --- | --- | --- | --- | --- | --- | --- | --- | --- | --- | --- | --- | --- |
| 2 | 55 | F | G12D | G12D (UHS) | 0.07 | 3 | 3.9x3.4x1.0 | 2a | Present | Present | 23.6 | lung | Deceased |
| 52 | 57 | F | G12D | G12D (UHS) | 0.75 | 3 | 4x 3.2x 3 | 1b | Absent | Present |  |  | F/U loss |
| 86 | 69 | M | G12V | G12V (UHS) | 0.13 | 3 | 1.2x 1x 0.8 | 0 | Absent | Absent | 31.8 | Lung, liver | Alive |
| 105 | 66 | F | G12C | G12C (Multiplex) | 0.19 | 3 | 4.5 x 3.5 x 1.9 | 2b | Absent | Absent | 17.6 | liver | Alive |
| 108 | 45 | M | G12S | G12S (Multiplex) | 0.58 | 3 | 11.0 x 4.0 x 3.5 | 2b | Present | Present | 16.6 | Retroperitoneum, lung, adrenal gl. | Deceased |
| 116 | 39 | F | G13D | G13D (UHS) | 0.48 | 3 | 8.2 x 4.5 x 1.3 | 2b | Absent | Absent | 23.6 | retroperitoneum | Alive |
| 135 | 55 | F | G13D | G13D (UHS) | 0.65 | 4b | 3.5 x 3.5 x 2.5 | 2b | Present | Present | 16.1 | Lung | Alive |
| 155 | 55 | F | G12V | G12V (UHS) | 1.18 | 3 | 5 x 4.5 x 2.7 | 1b | Present | Present | 13.6 | Lung | Alive |
| 156 | 58 | F | G13D | G13D (Multiplex, UHS) | 0.84 | 3 | 6.0 x 3.5 x 3.0 | 2b | Present | Present | 13.7 | Lung | Alive |

Abbreviations: LVI, lymphovascular invasion; PNI, perineural invasion; DFS, disease free survival

**Table G in S1 File. Comparison with digital droplet polymerase chain reaction (ddPCR) results (n=23)**

| sample | sample Qubit conc. | Input  DNA (ul) | Mutation types, tissue | MassARRAY technique. | ddPCR  (cut-off : 2 copy) |
| --- | --- | --- | --- | --- | --- |
| 1 | 0.32 | 3 | G12V (GGT-GTT) | G12V | Wild |
| 2 | 0.32 | 3 | G12C (GGT-TGT) | Wild | Wild |
| 3 | 0.33 | 3 | G12D (GGT-GAT) | G12D | Invalid |
| 4 | 0.34 | 3 | G12S (GGT-AGT) | G12S | Invalid |
| 5 | 0.31 | 3 | G12D (GGT-GAT) | G12D | Wild |
| 6 | 0.58 | 2 | G12D (GGT-GAT) | Wild | Wild |
| 7 | 0.60 | 2 | G12V (GGT-GTT) | G12V | G12X* |
| 8 | 0.58 | 2 | G12C (GGT-TGT) | G12C | Invalid |
| 9 | 0.66 | 2 | G12V (GGT-GTT) | G12V | Wild |
| 10 | 0.50 | 2 | G12D (GGT-GAT) | G12D | G12X |
| 11 | 0.85 | 1.5 | G12A (GGT-GCT) | Wild | Invalid |
| 12 | 0.71 | 1.5 | G12V (GGT-GTT) | G12V | G12X |
| 13 | 0.73 | 1.5 | G12D (GGT-GAT) | G12D | G12X/G13D |
| 14 | 0.87 | 1.5 | G12D (GGT-GAT) | G12D | G12X |
| 15 | 0.85 | 1.5 | G12V (GGT-GTT) | *G12V(UHS only)* | Wild |
| 16 | 0.91 | 1 | G12V (GGT-GTT) | *G12V(UHS only)* | Wild |
| 17 | 0.94 | 1 | G13D (GGC-GAC) | *G13D(UHS only)* | Wild |
| 18 | 1.47 | 1 | G12V (GGT-GTT) | G12V | G12X |
| 19 | 1.46 | 1 | G12V (GGT-GTT) | G12V | G12X |
| 20 | 1.55 | 1 | G12C (GGT-TGT) | Wild | Wild |
| 21 | 2.12 | after dilution_1 | G12A (GGT-GCT) | Wild | Wild |
| 22 | 7.90 | after dilution_1 | G12D (GGT-GAT) | *G12D(UHS only)* | Wild |
| 23 | 10.90 | after dilution_1 | G12V (GGT-GTT) | Wild | Wild |

* Specific amino acid could not be determined.

**Table H in S1 File. Clinical Characteristics and Mutation Analysis of 17 Patients with Additional Blood Samples**.

| Case ID | Group | Age | Sex | Mutation types, tissue | pT | Initial tumor size (cm ) | pN | Initial blood sample | | Additional blood sample | | | | DFS time  (m) | Metastisis site | Status |
| --- | --- | --- | --- | --- | --- | --- | --- | --- | --- | --- | --- | --- | --- | --- | --- | --- |
|  |  |  |  |  |  |  |  | cfDNA concentration (ng/μl) | Mutation analysis (type) | cfDNA concentration (ng/μl) | Mutation analysis (type) | Interval (m) | Event |  |  |  |
| 16 | Non-M | 58 | F | G13D | 3 | 4.3x 3.7x 1.3 | 1b | 0.64 | Wild | 0.16 | Mutant (G13D) | 60 | Metastasectomy | 18 | lung | A |
| 63 | Non-M | 59 | F | G13D | 2 | 1.3x 1.1x 0.6 | 0 | 0.20 | Wild | 0.75 | Mutant (G13D) | 22 | Metastasectomy | 16 | lung, liver | A |
| 70 | Non-M | 69 | M | G12D | 3 | 7.3x 5.8x 1.3 | 0 | 0.45 | Wild | 0.80 | Mutant  (G12D) | 14 | Metastasectomy | 13 | liver | D |
| 114 | M | 61 | F | G12D | 4a | 4 x 3 x 0.6 | 1b | 1.54 | Wild | 0.38 | Mutant  (G12D) | 20 | Metastasectomy | 0 | Ovary, lung, liver | D |
| 1 | Non-M | 67 | M | G12V | 3 | 7.5x 2.7x 2.5 | 0 | 0.18 | Wild | 0.13 | Wild | 37 | Metastasectomy | 17 | lung | D |
| 2 | Non-M | 55 | F | G12D | 3 | 3.9x 3.4x 1 | 2a | 0.07 | Mutant (G12D) | 0.17 | Wild | 25 | Metastasectomy | 24 | lung | D |
| 6 | Non-M | 71 | F | G12D | 3 | 4.2x 3.8x 1.2 | 2b | 0.17 | Wild | 0.20 | Wild | 5 days | follow-up | 22 | liver | D |
| 8 | M | 44 | M | G12V | 3 | 5.4x 4.3x 2.5 | 1b | 0.18 | Wild | 0.36 | Wild | 18 | Metastasectomy | 0 | liver | D |
| 38 | Non-M | 54 | M | G13D | 3 | 2.5x 1.7x 1.2 | 1b | 0.27 | Wild | 0.41 | Wild | 21 | Metastasectomy | 18 | lung | A |
| 43 | M | 59 | F | G12D | 3 | 2.5x 2.2x 1.6 | 0 | 1.05 | Wild | 0.17 | Wild | 12 | Metastasectomy | 0 | lung | loss |
| 45 | M | 51 | M | G12D | 3 | 6.9x 5.7x 3.6 | 1a | 1.99 | Mutant (G12D) | 0.34 | Wild | 5 | Metastasectomy | 0 | liver | A |
| 47 | M | 59 | M | G12V | 3 | 5x 3.5x 1 | 1b | 1.58 | Mutant (G12V) | 0.98 | Wild | 2 | Metastasectomy | 0 | lung | D |
| 69 | M | 60 | M | G12V | 4a | 6x 5.3x 1.1 | 1b | 0.34 | Mutant (G12V) | 1.15 | Wild | 23 | Metastasectomy | 0 | liver | D |
| 106 | Non-M | 62 | F | G12C | 3 | 5.0 x 4.0 x 1.0 | 0 | 0.11 | Wild | 0.30 | Wild | 19 | Metastasectomy | 19 | liver | A |
| 109 | M | 57 | M | G12D | 3 | 7.8 x 7.5 x 1.6 | 0 | 1.64 | Mutant (G12D) | 0.60 | Wild | 11 | Metastasectomy | 0 | liver | A |
| 124 | M | 62 | M | G12V | 2 | 3.5 x 2.5 x 1.0 | 0 | 0.71 | Wild | 0.38 | Wild | 22 | Metastasectomy | 0 | liver | A |
| 145 | Non-M | 33 | F | G12D | 3 | 5.0 x 4.5 x 1.4 | 1a | 0.35 | Wild | 0.63 | Wild | 21 | Metastasectomy | 20 | ovary | A |

Abbreviations: Non-M, non-metastasis; M. metastasis; status, survival status; A, alive; D, deceased; loss, follow up loss; DFS, disease free survival

**Table I in S1 File. Relative cost and number of mutations of mass spectrometry, ddPCR, and ultra-deep NGS.**

| Features | Mass spectrometry | ddPCR | Ultra-deep NGS |
| --- | --- | --- | --- |
| No. mutations | 73 hotspot mutations in four genes (*KRAS, NRAS, BRAF*, and *PIK3CA)* | 4 hot spot mutations of *KRAS* | 73 hotspot mutations in four genes (*KRAS, NRAS, BRAF*, and *PIK3CA)*: equivalent to 13 amplicons w/ 200bp size, mean target coverage 6,000× |
| Approximate cost / sample* | 25 USD | 25 USD | 130 USD |
| Relative cost | 1× | 1× | 5.2× |

*Costs only include charges for reagents and other supplies and does not include charges for labor or equipment.

USD: US dollars
